# Quantitative Approach for the Analysis of Fusional Convergence Using Eye-Tracking and SacLab Toolbox

**DOI:** 10.1155/2018/3271269

**Published:** 2018-07-22

**Authors:** Laura Cercenelli, Michela Fresina, Barbara Bortolani, Guido Tiberi, Giuseppe Giannaccare, E. C. Campos, Emanuela Marcelli

**Affiliations:** ^1^Laboratory of Bioengineering, Department of Experimental Diagnostic and Specialty Medicine (DIMES), Department of Biomedical and Neuromotor Sciences (DIBINEM), University of Bologna, Bologna, Italy; ^2^Ophthalmology Unit, Department of Experimental Diagnostic and Specialty Medicine (DIMES), University of Bologna, S. Orsola-Malpighi Teaching Hospital, Bologna, Italy; ^3^Laboratory of Bioengineering, Department of Experimental Diagnostic and Specialty Medicine (DIMES), University of Bologna, Bologna, Italy

## Abstract

Fusional vergence is a disjunctive movement of the eyes that is made in order to obtain single vision. The aim of the study was to provide a quantitative and objective approach for analyzing the fusional convergence response using eye tracking (ET) technology and automatic data analysis provided by the intuitive SacLab toolbox previously developed by our group. We evaluated the proposed approach in a population of 26 subjects with normal binocular vision, who were tested with base-out prisms (magnitudes 4Δ, 6Δ, and 10Δ) in order to elicit fusional convergence response. Eye movements were recorded using the Viewpoint ET and analyzed using SacLab. Parameters describing both the vergence and the version components of the fusional response (convergence duration, CD; peak convergence velocity, PCV; number of intrusive saccades, NS; and mean saccadic amplitude, MSA) were automatically calculated and provided to clinicians for an objective evaluation. Results showed that the number of subjects achieving fusional convergence decreased with prism magnitude. For subjects achieving fusion CD and PCV increased significantly (*p* < 0.05) when increasing the prism magnitude. For NS and MSA, there were no significant changes when passing to 6Δ, but a significant increase resulted when passing to 10Δ (*p* < 0.05). Noninvasive ET associated with the intuitive SacLab toolbox may represent a valid option to objectively characterize the fusional vergence response in clinical setting. The analysis may be extended to patients with vergence disorders.

## 1. Introduction

To binocularly view an object of interest, two different types of eye movements are used: saccades and vergence. Saccades are rapid conjugate (version) movements used to orient the eyes toward a new direction by rotating the two eyes with similar angles.

Vergence movements are produced when the eyes move through equal angles in opposite directions to bring the focal point of binocular gaze to different viewing distances in depth, by disjunctively rotating the eyes. This ensures that the projection of images on the retina of both eyes are registered with each other, allowing the brain to fuse the images into a single perception in order to provide stereoscopic vision of three-dimensional space [1, 2]. In horizontal vergence, when the lines of sight move inwards the eyes converge and when the line of sight move outwards, the eyes diverge.

Disorders in fusional vergence are often associated with the most common forms of strabismus. Patients with congenital esotropia have suboptimal vergence responses to fusional disparity stimuli, which remain to be fully characterized [3–7].

Fusional vergence ranges are traditionally determined by manipulating retinal disparity with occlusion and prisms [8–11]. While these evaluations are typically made using direct observation (therefore they may be quite subjective), an objective eye-tracking (ET) approach was proposed in this study. In recent years, the interest for noninvasive video-based ET systems to study and quantify eye movements and gaze patterns has increasingly grown [12, 13]. ET systems allow for objective eye movement recordings that may give important adjunct in diagnosis if compared with the routinely used qualitative evaluations, since more sensitive metrics and more specific testing paradigms for classes of patients can be provided [14–17].

The aim of the study was to provide a quantitative and objective approach for analyzing the fusional convergence response using ET technology and automatic data analysis provided by SacLab, a toolbox that we have previously developed to ease the use of ET systems in the clinical practice. We evaluated the proposed approach in a preliminary population of subjects with normal binocular vision.

## 2. Methods

### 2.1. Participants

The study group included 26 subjects (10 males, 16 females; age within the range 23–89 years, mean: 58 ± 23 years) with normal binocular vision: 10 were emmetropic or emmetropized by contact lenses, and 16 were presbyopic functionally emmetropic subjects. Functional emmetropia was defined as uncorrected visual acuity better than 20/20 in both eyes and uncyclopleged spherical equivalent refraction between +1 and −1 diopters.

The study was approved by the Ethics Committee of S. Orsola-Malpighi Hospital, Bologna; the procedures were in accordance with ethical practice, and participants signed a written consent.

Subjects with diseases that could affect fusional vergence capability (e.g., strabismus or ptosis, amblyopia, convergence insufficiency, glaucoma, and significant retinal pathology) were excluded from the study.

Binocular vision stereoacuity was evaluated with TNO stereotest and visual acuity at close distance (35 cm) with the Jaeger eye chart.

For each participant, eye dominance was determined with the hole-in-the-card test (Dolman method).

In the study group, 16 subjects were right eye dominant and 10 were left eye dominant.

### 2.2. Materials and Procedure

During the prism test, subjects were asked to wear the specially designed glasses (EyeFrame) equipped with infrared cameras and illuminators of the head-mounted ViewPoint ET system (sampling frequency: 60 Hz; accuracy: 0.25°–1.0° visual arc; spatial resolution: 0.15° visual arc, monocular/binocular tracking capability) by Arrington Research, Scottsdale, AZ, USA. Subjects were seated in front of a LCD, with their heads supported by a chin rest and blocked with a removable elastic band to avoid unwanted head movements. For each participant, the ET equipment was calibrated in monocular vision, asking the subject to fixate central and peripheral targets (16 points calibration). Then, the subject underwent the previously prepared prism test to elicit horizontal fusional convergence, while eye movements were captured with the ET system. Data recording and analysis were performed using SacLab, an intuitive Matlab toolbox that we previously developed to analyze eye movements recorded with the Arrington ViewPoint ET system [18].

The prism test is the most commonly used test to induce disparity to evaluate the fusional capability when the binocular vision is regained through the prism. The visual stimulus used for the prism test was presented on a 19″ LCD, at a viewing distance of 40 cm. The visual stimulus consisted of a black dot (fixation point) on a white background, occupying 0.5° of the subject's field of view, surrounded by red arrows pointing to it and located at the center of the LCD.

Subjects were asked to keep their eyes on the fixation point; after 5 seconds of fixation, the operator inserted a base-out prism in front of the dominant eye in order to elicit a horizontal fusional convergence movement. Subjects were asked that, in case of double vision, they should try to fuse the images from both eyes and maintain a clear view of the fixation point. The prism was removed after 5 seconds from insertion. For each subject, the prism tests were carried out using 4Δ, 6Δ, and 10Δ prisms that were presented in a random order. For each prism, a minimum of 5 repetitions of the same test were performed. Before changing the prism, the subjects were allowed to rest for about 30 seconds.

### 2.3. Quantification of Fusional Response

Generally, the monocular placement of a base-out prism elicits a vergence response stimulated by the experienced diplopia, which takes the form of a convergence movement performed by the subject in order to achieve single vision. As mentioned in previous studies [19–22], saccade intrusive components occur naturally during vergence even when a pure vergence movement is elicited. In the majority of cases, these small saccades occur either at the onset of the convergence or later during the convergence phase [22].

To study the fusional response elicited by the prism insertion, we based our quantitative approach on the analysis of both the vergence (convergence) and the version (saccade) components.

**Figure 1:**
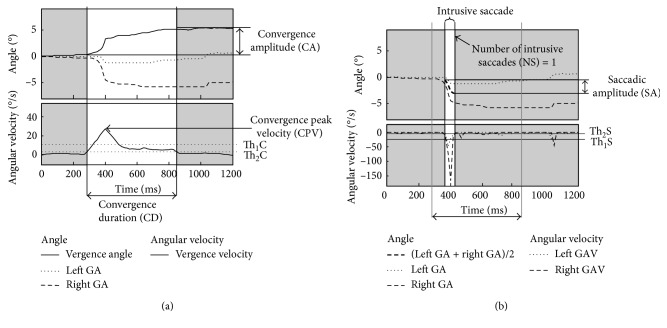
Example of eye movement recordings with ET system and automatic analysis implemented in SacLab to derive the descriptive parameters for fusional response. (a) Analysis of vergence response: identification of convergence using a threshold-based algorithm (Th_1_C, Th_2_C) and calculation of convergence parameters (CD = convergence duration; CA = convergence amplitude; and CPV = convergence peak velocity). (b) Analysis of version response: identification of intrusive saccades using a threshold-based algorithm (Th_1_S, Th_2_S) and calculation of saccadic parameters (NS = number of intrusive saccades; MSA = mean saccadic amplitude).

For the version component, we considered the saccades occurring together with the convergence movement, that we defined “intrusive” saccades. An example of fusional convergence, together with an intrusive saccade, is illustrated in Figure 1(b). For each eye, the azimuth component of gaze angle (GA) was recorded using the ViewPoint ET system. We introduced these following parameters describing the fusional response that we calculated using semiautomated algorithms developed in MATLAB (Mathworks, Natick, MA, USA) and implemented in the SacLab toolbox.

#### 2.3.1. Vergence Response: Parameters for Convergence

The instantaneous horizontal vergence response (vergence angle) was calculated by subtracting the horizontal angular position of the left eye (left GA) from that of the right eye (Right GA), as widely used in the field of ophthalmology (Figure 1) [3, 22–25]. Using this convention, the vergence angle increases during convergence movements and decreases during divergence movements.

Through numerical differentiation of the vergence angle, the rate of change of vergence (vergence velocity) was calculated (Figure 1). Vergence velocity (positive for convergence movements and negative for divergence movements) was used to identify convergence, via a threshold-based algorithm similar to the one we have previously described for saccade recognition with Saclab toolbox [18]. We defined two threshold values: a first threshold (Th_1_C = 10°/s) used for peak detection in the vergence velocity signal; a second threshold (Th_2_C = 1°/s) to define a baseline value. Then, the following parameters were automatically calculated to describe convergence (Figure 1(a)): *Convergence Duration (CD)*, defined as the duration of the convergence phase: the time section in which vergence velocity rises from the baseline (Th_2_C), reaches a peak exceeding Th_1_C, and drops below the baseline again; *Convergence Amplitude (CA)*, defined as the maximum excursion of vergence angle within the convergence duration; *Convergence Peak Velocity (CPV)*, defined as the maximum vergence velocity during the convergence duration.

#### 2.3.2. Version Response: Parameters for Intrusive Saccades

The intrusive saccades were identified using a threshold-based algorithm with predefined threshold values (Th_1_S = 25°/s and Th_2_S = 3°/s) applied to the gaze angular velocity (GAV) signals of both eyes. The phase of an intrusive saccade was identified as the time section in which both the left and right GAV signals shift in the same direction from the baseline (Th_2_S), reach positive (or negative) peaks exceeding Th_1_S and drop below the baseline again (Figure 1(b)). The amplitude of an intrusive saccade (saccadic amplitude, SA) was defined as the maximum excursion of the average gaze angle ((Left GA + Right GA)/2) during the identified intrusive saccade phase.

The following parameters were automatically calculated to describe the version response (intrusive saccades): *number of intrusive saccades (NS)*; *mean saccadic amplitude (MSA)*, defined as the mean SA value for all of the detected intrusive saccades. If no intrusive saccades were identified, both parameters were set equal to zero.

### 2.4. Identification of Fusions

During eye movements recording, a preliminary data analysis was performed to discriminate if a subject was capable of achieving fusion: a real-time comparison between the analyzed vergence response and the prism magnitude (PM) used (converted in degrees) was carried out. The following criteria were used to identify a successful fusion: (1) convergence was recognized (i.e., vergence angle >0); (2) the calculated convergence amplitude (CA) was compatible with the deviation expected for the inserted prism (i.e., CA within the range PM ± 1°); (3) a stable convergence was maintained (i.e., vergence velocity remained below baseline threshold Th_2_C for at least 2 seconds).

When all these three criteria were verified, an acoustic feedback was given so that the clinician could know if the subject had achieved fusion (based on an objective evaluation), and the prism test was marked as the “fusion” test.

For each prism (4Δ, 6Δ, and 10Δ), a minimum of 5 tests were performed: if none of these tests was marked as “fusion,” the subject was considered incapable to achieve fusion with the presented prism. If at least one test was marked as “fusion,” the operator proceeded with additional prism tests in order to collect at least 3 “fusion” tests for each prism magnitude to be used for obtaining mean values for the calculated parameters.

### 2.5. Modality of Fusions: Effect of Prism Magnitude

This further analysis was addressed to explore the influence of prism magnitude on the fusion modality by analyzing possible changes of the calculated descriptive parameters for both the convergence response (CD and CPV) and the saccadic response (NS and MSA).

Only those subjects who achieved fusion with all prisms (4Δ, 6Δ, and 10Δ) were eligible for this analysis on fusion modality.

For each subject and for each prism magnitude, the descriptive parameters (CD, CPV, NS, and MSA) were reported as mean values over 5 “fusion” tests.

#### 2.5.1. Statistics

One way ANOVA was used to evaluate if the different prism magnitude has an effect on the calculated descriptive parameters. Then, to further explicate the group differences that contribute to significance, a post-ANOVA test for multiple comparison analysis (i.e., Student–Newman–Keuls, SNK test) was applied.

A *p* level of 0.05 was chosen. Statistical analysis was performed in SPSS (IBM SPSS, New York, NY).

## 3. Results

### 3.1. Results on Fusion Identification

**Figure 2:**
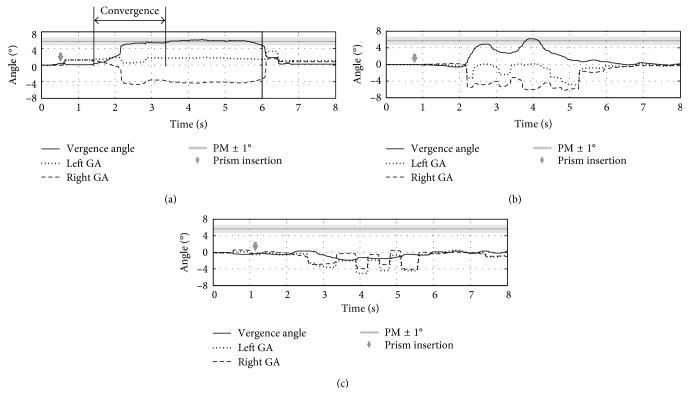
Examples of ET recordings: (a) successful “fusion”: convergence occurs (vergence angle >0); convergence amplitude (CA) falls in the range of PM ± 1°; stable convergence is maintained for more than 2 seconds. (b) “Failed” fusion: attempt to fuse, but CA is not maintained. (c) “Failed” fusion: rapid switching of both eyes from one position to another, without ever reaching the expected CA. For all tests the inserted prism was 10Δ.

Representative traces of eye movement recordings during the prism tests are reported in Figure 2.

The first trace (Figure 2(a)) shows a typical response of a subject who achieves fusional convergence (marked as the “fusion” test), since there is a clear convergence movement (vergence angle >0), the calculated CA is in the range of PM ± 1° (considering conversion of PM in deviation angles, i.e., 5.71° for 10∆) and the CA value is maintained for more than 2 seconds.

The second trace (Figure 2(b)) shows an attempt to fuse, where the expected CA is achieved, but it is not maintained for more than 2 seconds.

Finally, the third trace (Figure 2(c)) shows a case of complete absence of convergence, where rapid switching of both eyes from one position to another occurs, without ever reaching the expected CA.

**Figure 3:**
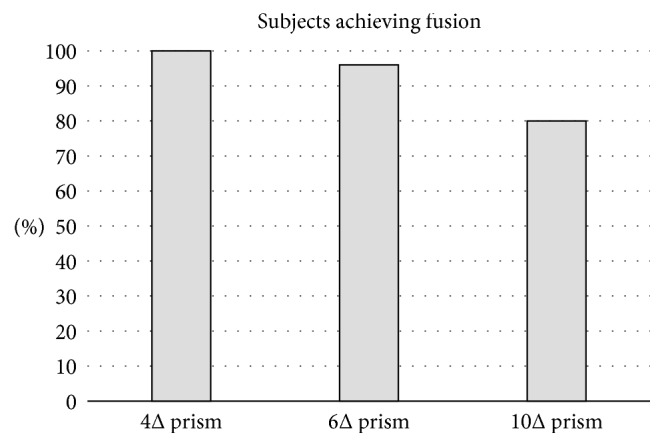
Percentage of subjects achieving fusion for different prism magnitudes, as provided by the implemented automatic analysis.

In our analysis, both the attempts to fuse (Figure 2(b)) and the complete absence of convergence (Figure 2(c)) were classified as “failed” fusions. By collecting results on fusion identification, we found that the number of subjects achieving fusional convergence decreased with the magnitude of the prism inserted (Figure 3).

### 3.2. Results on Fusion Modality

The dataset for this analysis included a subgroup of 19 subjects (8 males, 11 females, 54 ± 23 years), that is, those who achieved fusion with all the presented prisms. For each subject, fusion modality was automatically analyzed by calculating the descriptive parameters for convergence and saccadic response (CD, CPV, NS, and MSA) and averaging them over the 5 “fusion” tests resulting from each prism.

**Figure 4:**
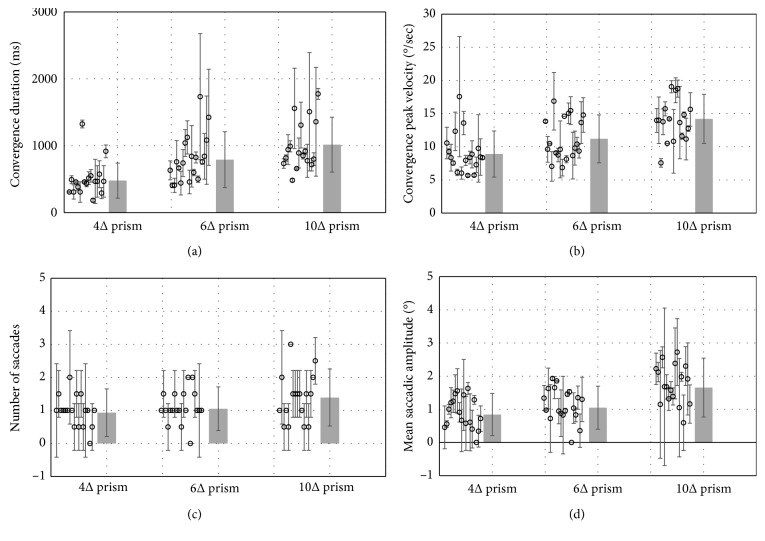
Analysis of fusion modality over different prism magnitudes: variations of descriptive parameters (mean ± SD) for vergence (convergence duration: CD; convergence peak velocity: CPV) and saccadic response (number of intrusive saccades: NS; mean saccadic amplitude: MSA). The histogram bars represent mean values, while the white dots represent the individual vergence response and standard errors in the sample (each dot is the mean value of repeated measurements for each subject in the same experimental condition).

All the descriptive parameters showed an increasing trend when the prism magnitude was increased (Figure 4).

There was a statistically significant difference between means of the calculated descriptive parameters for the three groups of prism magnitude (4Δ, 6Δ, and 10Δ), as determined by ANOVA analysis (*F*(3.07) = 21.755, *p* < 0.001; *F*(3.07) = 22.354, *p* < 0.001; *F*(3.07) = 4.194, *p* < 0.001, *F*(3.07) = 13.638, *p* < 0.001, for CD, CVP, NS, and MSA, resp.).

SNK post-ANOVA test for multiple comparison revealed that the convergence parameters (CD and PCV) varied significantly (*p* < 0.05) when increasing the prism magnitude ranged from 4Δ to 6Δ, from 6Δ to 10Δ, and from 4Δ to 10Δ (Table 1). For saccadic parameters (NS and MSA), there was no significant changes when passing from 4Δ to 6Δ, but a significant increase resulted when passing to 10Δ (Table 1).

## 4. Discussion

In this study, we presented a quantitative ET-based approach to analyze the fusional convergence response in subjects with normal binocular vision.

The implemented real-time analysis of ET recordings allowed to easily and quickly identify the fusion responders for the used prism, instead of relying on subjective participant's feedback.

As interesting advancements following our preliminary ET-based analysis of fusional convergence [26, 27], we introduced the calculation of parameters that provide quantitative description of both the vergence (CD and CPV) and the version components (NS and MSA) typically occurring during a disparity-driven test performed to elicit the fusional vergence response.

Our quantitative analysis showed that the ability to achieve fusion decreased when the size of the elicited disparity (prism magnitude) increased. Additionally, the subgroup of subjects achieving fusion showed different modalities of fusion when varying the prism magnitude (PM). For the convergence component, we found that convergence duration (CD) was longer with increasing PM, as well as the peak convergence velocity (PCV) was higher.

Prolongation of time to reach stable convergence amplitude (increased CD) may be related to the fact that when PM increases, it becomes more difficult to fuse the stimulus image.

In agreement with previous observations [22, 25], the increase of CPV may represent a mechanism to facilitate (to accelerate) the fusion when high disparities are induced, that is, high PM are applied. As concerning the mean values of CPV, our findings for prism magnitude of 10∆ (about 5° of disparity) were comparable with maximum convergence velocities reported by an earlier research of Alvarez et al. [28] that studied and tracked 4° disparity step changes for convergence and divergence.

For the version component, we found that the number of intrusive saccades has a tendency to increase when PM increases. Also, these findings seem to fit with previous observations on normal fusional vergence eye movements [21, 22, 24, 25, 29, 30], that documented how most subjects made horizontal small saccades when targets were set to elicit only vergence. These previous studies proposed the existence of strong interactions between the saccadic and the vergence subsystem, and they all found that combining version movements to vergence shifts might facilitate the vergence response. Particularly, Van Leeuwen et al. [31, 32] reported that when small saccades were associated with vergence shifts, the vergence peak velocities were usually higher than during pure vergence shifts. Therefore, a possible function of a saccade intrusive component could be the enhancement of vergence, so that a new target is fixated more quickly [32].

In our study, we applied the proposed quantitative approach for the analysis of the binocular response to monocular placement of prisms in front of the dominant eye. Some previous studies performed similar tests and found that there are many different responses comprising variations of vergence and saccadic movements [19, 33, 34]; however, they did not provide a detailed analysis based on descriptive parameters, as the one we provided by the presented approach.

### 4.1. Study Limitations

Following the clinical practice, this study focuses on vergence response induced by prisms. Indeed, prisms stimulate only the fusional component of the vergence; therefore, the accommodative component is not taken into account.

The power of the prisms evaluated in this study is rather small (up to 10 diopters). However, according to the objective recording of vergence eye movements, some of the subjects were not able to fuse. This sounds a bit surprising since all subjects were screened to exclude pathology including convergence insufficiency. This discrepancy between results of objective recordings and clinical testing should be further investigated in future studies.

In this study, the application of the quantitative approach was limited to fusional convergence response, while the divergence movement is not analyzed. To fully characterize the fusional vergence response in subjects with normal binocular vision, we should extend our analysis to fusional divergence response, using base-in prisms.

As previously investigated by other researchers [35–37], we could expand our current analyses based on an objective eye tracker method with additional calculations of fixation disparity at the end of the vergence step response.

Following our previous observations [27], additional tests could be planned to apply our approach for evaluating if presentation of disparity visual stimulus on textured backgrounds, rather than on white background, can facilitate the fusional response.

Future activities will also be addressed to extend the described ET-based quantitative analysis to patients with disorders in fusional vergence, particularly subjects with the most common forms of strabismus.

## 5. Conclusion

The present study showed that noninvasive ET associated with the intuitive SacLab toolbox may represent a valid option to objectively characterize the disparity-driven fusional convergence response in clinical setting. The results collected in this preliminary study population may contribute for better characterization of the fusional convergence response in normal subjects.

The proposed quantitative approach may provide objective diagnostic criteria to be used by clinicians to identify fusional vergence disorders, as well as to quantify improvements in the fusion capability after surgical treatments, with interesting clinical implications.

## Figures and Tables

**Table 1:** Results of SNK post-ANOVA analysis to evaluate the effect of prism magnitude.

Prism magnitude	CD		CPV		NS		MSA	
	Mean ± SD (ms)	SNK	Mean ± SD (°/s)	SNK	Mean ± SD	SNK	Mean ± SD (°)	SNK
4Δ	478 ± 262	a	8.91 ± 3.47	a	0.93 ± 0.72	a	0.84 ± 0.64	a
6Δ	792 ± 418	b	11.20 ± 3.61	b	1.05 ± 0.66	a	1.05 ± 0.65	a
10Δ	1016 ± 410	c	14.20 ± 3.71	c	1.39 ± 0.86	b	1.66 ± 0.89	b

CD, convergence duration; CVP, convergence peak velocity; NS, number of intrusive saccades; MSA, mean saccade amplitude. Means followed by the same letter in the column do not differ statistically (*p*=0.05).

## Data Availability

The datasets generated and/or analyzed during the current study are available from the corresponding author on reasonable request.
